# Accurate diagnosis of breast lesions: Number 4 – April 2023

**DOI:** 10.1055/s-0043-1769468

**Published:** 2023-05-24

**Authors:** Alexandre Vicente de Andrade, Clécio Ênio Murta de Lucena, Danielle Chambô dos Santos, Eduardo Carvalho Pessoa, Fabio Postiglione Mansani, Felipe Eduardo Martins de Andrade, Giuliano Tavares Tosello, Henrique Alberto Portella Pasqualette, Henrique Lima Couto, Jose Luis Esteves Francisco, Rodrigo Pepe Costa, Sandra Regina Campos Teixeira, Thaís Paiva Moraes, Agnaldo Lopes da Silva Filho

**Affiliations:** 1 Pontifícia Universidade Católica de São Paulo, São Paulo, SP, Brazil.; 2 Universidade Federal de Minas Gerais, Belo Horizonte, MG, Brazil.; 3 Faculdade de Medicina da Santa Casa de Misericórdia de Vitoria, Vitória, ES, Brazil.; 4 Universidade Estadual Paulista, Botucatu, SP, Brazil.; 5 Universidade Estadual de Ponta Grossa, Ponta Grossa - PR, Brazil.; 6 Hospital Sírio-Libanês, São Paulo, SP, Brazil.; 7 Universidade do Oeste Paulista, Presidente Paulista, SP, Brazil.; 8 Centro de Pesquisas da Mulher Rio de Janeiro, Rio de Janeiro, RJ, Brazil.; 9 Clínica Redimama-Redimasto de Belo Horizonte, Belo Horizonte, MG, Brazil.; 10 Faculdade de Medicina de São José do Rio Preto, São José do Rio Preto, SP, Brazil.; 11 Hospital de Base do Distrito Federal, Brasília, DF, Brazil.; 12 Pontifícia Universidade Católica de São Paulo, Campinas, SP, Brazil.; 13 Rede Mater Dei de Saúde, Belo Horizonte, MG, Brazil.; 14 Universidade Federal de Minas Gerais, Belo Horizonte, MG, Brazil.

## Key points

- Breast lesions comprise a wide variety of diagnoses with different manifestations.
- Breast lesions can be classified as benign, of uncertain malignant potential (B3), carcinoma in situ, and invasive carcinoma.
- In the era of personalized medicine, individualizing and getting an accurate diagnosis makes a big difference in the patient’s final outcome, especially in the case of breast cancer.
- Targeted and quality imaging exams, properly selected biopsy methods and conventional anatomopathology, immunohistochemistry and even molecular analyzes are crucial in the diagnosis and management of patients.

## Recommendations

- The minimal imaging propaedeutics indicated in the assessment of breast lesions is mammography and ultrasound of the breasts and armpits, which are sufficient in most cases.
- The diagnosis of patients with palpable lesions of suspicious characteristics on clinical examination should not be delayed; therefore, core biopsy should be indicated, preferably ultrasound-guided core biopsy.
- For suspicious lesions detected by imaging tests, the choice of biopsy method should consider the presentation and size of the lesion and in which imaging methods the lesion is visualized.
- Whenever the lesion is visualized on ultrasound, this will be the method of choice to guide the minimally invasive procedure.
- When the lesion is visible on magnetic resonance imaging (MRI) of the breasts, a second look ultrasound should be performed in an attempt to find the lesion. Mammography with localized compression and magnification can also be performed, especially in the case of non-mass enhancements in an attempt to localize the lesion. Second look tomosynthesis considerably increases lesion localization rates. If no other method can visualize the suspicious finding, the biopsy should be MRI-guided.
- Every service that proposes to offer MRI as a screening option must have means for performing MRI-guided biopsy in its own service or in a referenced service. Alternatively, preoperative marking methods for performing a surgical biopsy can be performed. However, performing therapeutic procedures without prior knowledge of the nature of the lesion is not allowed.
- In the case of suspicious mass lesions (nodules) larger than 1 cm, core biopsy should be the preferred method of biopsy. In nodules smaller than 1 cm, both core biopsy and vacuum biopsy may be indicated, depending on the individual case.
- In complex solid-cystic lesions with a solid component smaller than 1 cm, vacuum-assisted biopsy (VAB) should be indicated preferably. In lesions with an extensive solid component, core biopsy or VAB can be used, depending on availability and the degree of suspicion.
- In polypoid intraductal lesions (suspected papilloma), VAB should be indicated as a diagnostic method, if available.
- For lesions that present as architectural distortion and probable radiating scar, VAB is more accurate than core biopsy.
- For microcalcifications seen only on mammography, stereotactic vacuum biopsy should be the method of choice whenever available.
- In lesions of uncertain malignant potential (B3) or in cases of inconclusive core biopsy, vacuum-assisted excision (VAE) is indicated.
- The recommendation for probably benign BI-RADS category 3 lesions is the biannual follow-up in the first year, and annual follow-up thereafter. In patients younger than 30 years old with BI-RADS category 3 nodules, simple or complicated cysts, fine needle aspiration biopsy (FNAB) is indicated if desired by the patient or when indicated by the clinician.
- In the case of suspected axillary lymph nodes, the evaluation can be performed by FNAB or core biopsy according to the indication and evaluation of the case. The location of these lymph nodes and the expertise of the physician performing the examination should also be considered.
- A pathologist experienced in breast pathology is imperative for pathological evaluation and accurate diagnosis.
- Immunohistochemical panel should be mandatory for all cases of ductal carcinoma in situ and invasive carcinoma.
- Immunohistochemistry should be performed whenever the pathologist deems it necessary, being essential in cases of malignant breast lesions.
- Immunohistochemical panel of breast carcinoma is prognostic, predictive and should include estrogen receptor, progesterone receptor, HER2/neu and Ki 67.
- Molecular exams and prognostic genetic panels have specific indication and are tools that can contribute in selected cases.
- Clinical, imaging and pathology agreement is essential for the accurate diagnosis. It is the role of the physician who performs outpatient diagnostic procedures to be aware of the results, both for audit purposes and to exchange information with requesting physicians, if necessary.

## Background

Breast lesions comprise a wide variety of diagnoses with different behaviors and presentations. The broad aspect of suspicious breast lesions ranges from proliferative lesions without atypia to carcinomas. Breast lesions can be grouped into three categories that present different risks and management: benign lesions of uncertain malignant potential (pathological classification B3), in situ carcinomas and invasive carcinomas. 1 2 3 A specific diagnosis is the goal of all investigation, but it is important to confirm malignancy or exclude it. In the era of personalized medicine, individualizing makes a big difference in case management. Targeted and high-quality imaging exams, properly selected biopsy methods and conventional anatomopathology, immunohistochemistry and even molecular analyzes can be decisive for the diagnosis and management of patients. The investigation of breast lesions can result from two different situations: screening or diagnosis. The interpretation of imaging findings, the indication of the biopsy technique, the interpretation of results and the correlation of clinical, imaging and pathology may vary depending on whether the finding is due to screening in asymptomatic patients or patients with complaints, signs or symptoms on physical examination in a diagnostic situation. 4 5

## Imaging propaedeutics

Anamnesis and complete clinical examination should be performed in patients with complaints and clinical alterations resulting from screening. In the case of clinically suspicious lesions, core biopsy should be indicated immediately, preferably ultrasound-guided core biopsy. Mammography and ultrasound are the minimum propaedeutics for the evaluation of breast lesions. 6 Mammography is not necessary in patients under 30 years of age, especially in those under 25 years of age with nodules suggestive of benign BI-RADS category 3. Tomosynthesis can be particularly useful in the assessment of breasts lesions with density pattern B (sparse areas of fibroglandular tissue) and C (heterogeneously dense) according to the BI-RADS classification. 6 7 Magnetic resonance imaging of the breasts can be used in selected cases and in patients at high risk for breast cancer; in its absence, contrast-enhanced mammography can be used. Thermography and other alternative imaging methods are still in the experimental phase, have not demonstrated any additional benefit in the diagnosis of breast lesions and currently have no indication in the investigation or diagnosis of breast lesions. The Ministry of Health and the National Cancer Institute (Inca) strongly recommend not incorporating thermography into the line of breast diagnostic care. 8 9 10 11 12

## What is the imaging method of choice to guide the biopsy procedure?

Although biopsies can be performed manually without an associated imaging method, this association improves the results, so it should always be used when available. Whenever the lesion is visualized on ultrasound, this is the method of choice to guide the procedure. For calcifications seen only on mammography, the method of choice is stereotaxis. For architectural distortions and focal asymmetries seen on mammography, methods of choice are tomo biopsy (tomosynthesis-guided biopsy) and in its absence, stereotaxis. Lesions seen only on tomosynthesis should be approached by tomo biopsy. 13 Lesions seen only on MRI should be biopsied using this method. Contrast-enhanced mammography biopsy is not available in our country. 14 15

## Is it a minimally invasive procedure?

The preferred biopsy method for solid lesions should be histological. 8 BI-RADS category 3 nodules and changes should be followed up every six months for one year and annually thereafter, except for probably benign BI-RADS category 3 lesions in patients younger than 30 years and simple or complicated cysts, in which cytology is well indicated when, for clinical reasons or patient concern, an invasive procedure has been requested. 15 Fine needle aspiration biopsy is performed to obtain cytological material in mastology. Core biopsy is performed using tru-cut mechanisms (elastic potential energy, spring) and 14 G to 18 G needles. Most lesions are satisfactorily diagnosed with core biopsy. 16 Vacuum biopsy rescues a fragment of the lesion by means of vacuum suction using 7G to 12G needles. 17 Both core biopsy and vacuum biopsy can be used in lesions smaller than 1.0 cm according to the individual case. 4 5 6 14 18 19 Vacuum-assisted biopsy is preferred in complex solid-cystic lesions with a solid component smaller than 1 cm, and core biopsy or VAB may be used in those with a solid extensive component, depending on availability and degree of suspicion. In polypoid intraductal lesions (suspected papilloma), VAB should be indicated as the diagnostic method, if available. 20 For microcalcifications seen only on mammography, stereotactic vacuum biopsy should be the method of choice, whenever available. 14 Diagnostic vacuum-assisted excision (VAE) is defined as the complete percutaneous excision of the lesion or the salvage of more than 4 g of tissue. 21 In lesions of uncertain malignant potential or cases of inconclusive core biopsy, VAE is indicated. 1 16 18 21 Incisional or excisional surgical biopsy is currently reserved for cases of clinical-imaging-pathological disagreement or situations in which percutaneous methods cannot be performed because of unavailability or technical contraindication, such as risk of pneumothorax. 14

## What is the pathology?

Breast pathology is a concentration field and requires targeted training. A pathologist experienced in cytopathology and breast pathology is imperative for cytological and pathological evaluation and accurate diagnosis. Most diagnoses can be confirmed in established tissue analysis using hematoxylin-eosin staining. Diagnostic immunohistochemistry should be performed whenever the pathologist deems it necessary and may be essential to confirm the diagnosis in some situations. The immunohistochemical panel is prognostic and predictive, and should be performed for all in situ or invasive breast carcinomas, as it allows approximating the molecular classification of invasive breast cancer, classifying breast tumors as luminal-like (tumors with hormone receptors, estrogen and progesterone positive), HER2-like (with expression of the HER2 membrane protein) and basal-like (tumors lacking hormone receptors and the HER2 membrane protein). Currently, immunohistochemical panel is used for decisions regarding all breast cancer therapy, since, in addition to being prognostic, it has predictive value for endocrine therapy, anti-HER2 therapy, chemotherapy and immunotherapy. 22 23

## Molecular tests

Molecular tests and prognostic genetic panels have a specific indication and should not be performed in a generalized way for all cases of malignancy.

However, doing these tests can optimize the treatment of many patients, either by adding or removing systemic treatments. Molecular tests allow individualizing each patient according to their specific risk and directing the best treatment.

## Final considerations

In the era of precision medicine and personalization of procedures, the accurate diagnosis of breast lesions is essential. The clinical situation of the patient (screening versus diagnosis), imaging tests, the biopsy technique used, the imaging method to guide the procedure, the cytological-histological-immunohistochemical diagnosis and eventually the molecular diagnosis must be taken into account for the proper diagnosis of breast lesions. Although all this arsenal is available, clinical agreement with imaging and pathology are essential as well. In the occurrence of any disagreement of findings, the case should be reviewed and a new biopsy should always be considered.

National Commission Specialized in Breast Imaging of the Brazilian Federation of Gynecology and Obstetrics Associations (Febrasgo)

President:

Eduardo Carvalho Pessoa

Vice-president:

Henrique Lima Couto

Secretary:

Clecio Enio Murta De Lucena

Members:

Alexandre Vicente de Andrade

Danielle Chambô

Fabio Postiglione Mansani

Felipe Eduardo Martins De Andrade

Giuliano Tavares Tosello

Hélio Sebastião Amâncio de Camargo Júnior

Henrique Alberto Portella Pasqualette

Jorge Villanova Biazús

José Luis Esteves Francisco

Rodrigo Pepe Costa

Sandra Regina Campos Teixeira

Thais Paiva Moraes
